# The role of Western diet and gut microbiota in the pathogenesis of cardiovascular diseases

**DOI:** 10.3389/fmicb.2026.1608563

**Published:** 2026-02-27

**Authors:** Ziman He, Bo Liu, Aimin Gong, Xiaokang Jia

**Affiliations:** 1 School of Traditional Medicine, Hainan Academy of Medical Sciences, Hainan Medical University, Haikou, Hainan, China; 2 Key Laboratory of Basic Pharmacology and Joint International Laboratory of Ethnic Medicine, Ministry of Education, Zunyi Medical University, Zunyi, China

**Keywords:** cardiovascular diseases, intestinal microbiota, regulation of the microbiota, trimethylamine-N-oxide, Western diet

## Abstract

The Western diet (WD) is characterized by high fat, high sugar, high salt and low fiber. WD can disrupt the homeostasis of the intestinal flora and become an important factor in the occurrence and development of Cardiovascular Diseases (CVD). This review elucidates the core mechanism through which WD-induced intestinal flora dysbiosis contributes to the development of CVD. Specifically, the disruption of intestinal barrier function promotes the generation of pathogenic metabolites, such as trimethylamine-N-oxide (TMAO), while simultaneously suppressing the production of beneficial metabolites, including short-chain fatty acids (SCFAs). This metabolic shift subsequently triggers systemic inflammatory responses, oxidative stress, and metabolic disturbances, thereby accelerating the progression of CVD-related conditions, such as atherosclerosis and hypertension. Meanwhile, this review systematically summarizes key intervention strategies targeting the gut microbiota. Accumulating evidence indicates that interventions such as probiotics, prebiotics, the Mediterranean diet, and fecal microbiota transplantation (FMT) can effectively restore intestinal microbial homeostasis, enhance the production of SCFAs, and mitigate the risk of CVD. Notably, long-term dietary patterns have demonstrated significant efficacy in reshaping the gut ecosystem, underscoring the importance of sustainable lifestyle modifications. Therefore, this study aims to integrate current knowledge regarding the underlying molecular mechanisms and provide a theoretical basis for developing precise interventions to prevent and treat CVD through modulation of the gut microbiota.

## 1 Introduction

Cardiovascular diseases (CVD) include coronary atherosclerotic heart disease, hypertension (HTN), hyperlipidemia, atrial fibrillation, heart failure (HF), myocardial infarction (MI), etc. CVD is the leading cause of death and premature death in China (Park and Park, 2015), accounting for about 40% of the total annual deaths (Lozano et al., 2012). The incidence of CVD can be effectively reduced by effective control of CVD risk factors such as smoking, alcohol abuse, sedentary lifestyle, unhealthy dietary habits, and obesity (Lozano et al., 2012).

The human gastrointestinal tract perched a by about 200 different kinds of bacteria, viruses and fungi community composition of diverse ecosystems, referred to as gut microbiome. These microbial populations play key roles in host physiological processes by providing essential metabolic functions, significantly influencing health maintenance and disease development. Studies have shown that suboptimal dietary patterns significantly modulate key aspects of gut microbiota homeostasis, including changes in gut pH levels, increased gut permeability, and dysregulation of bacterial metabolic byproducts (Mathers and Loncar, 2006; Lloyd-Jones et al., 2002). WD is mainly composed of highly processed and ultra-processed foods (Farchi et al., 1995; Ding et al., 2025; Freedman et al., 2003) and is one of the most typical suboptimal dietary patterns characterized by excessive intake of refined sugar, saturated fat, and sodium, along with insufficient intake of dietary fiber, especially from plant sources. A large number of studies have shown that WD can significantly alter the imbalance of the microbiota, disrupt the integrity of the intestinal barrier, and enhance intestinal permeability, thereby promoting the entry of toxic metabolites from microbial sources into the systemic circulation (Nettleton et al., 2007). Emerging evidence from recent epidemiological studies suggests that WD has reached epidemic scale worldwide. Long-term intake of WD can cause various pathological consequences, not only leading to intestinal flora imbalance, but also causing hyperinsulinemia, dyslipidemia, weakened systemic inflammatory response and endotoxemia. Together, these pathological changes will accelerate the occurrence of CVD, metabolic syndrome and gastrointestinal dysfunction (Oikonomou et al., 2018; Suh et al., 1992; Schwab et al., 2014).

Although studies have initially confirmed the association between WD, gut microbiota and CVD, there are still many key scientific problems to be elucidated: First, the differentiated regulatory effects and synergistic mechanisms of different components such as high sugar, high fat, and high salt in WD on the intestinal flora remain unclear. For instance, whether a high-sugar and high-fat diet will exacerbate the imbalance in the ratio of *Firmicutes* to Bacteroides through a superimposed effect; Secondly, there are differences in the specific action pathways of gut microbiota metabolites (such as short-chain fatty acids and trimethylamine-N-oxides) in different CVD subtypes (such as coronary heart disease and HF), and existing studies have insufficient analysis of the subtype-specific mechanisms of this. Thirdly, the influence of factors such as individual genetic background (such as APOE gene polymorphism) and the baseline composition of intestinal flora on the susceptibility to WD-related CVD has not yet formed a systematic understanding.

## 2 WD disrupts the balance of the intestinal microbiota in the body

**Figure 1:**
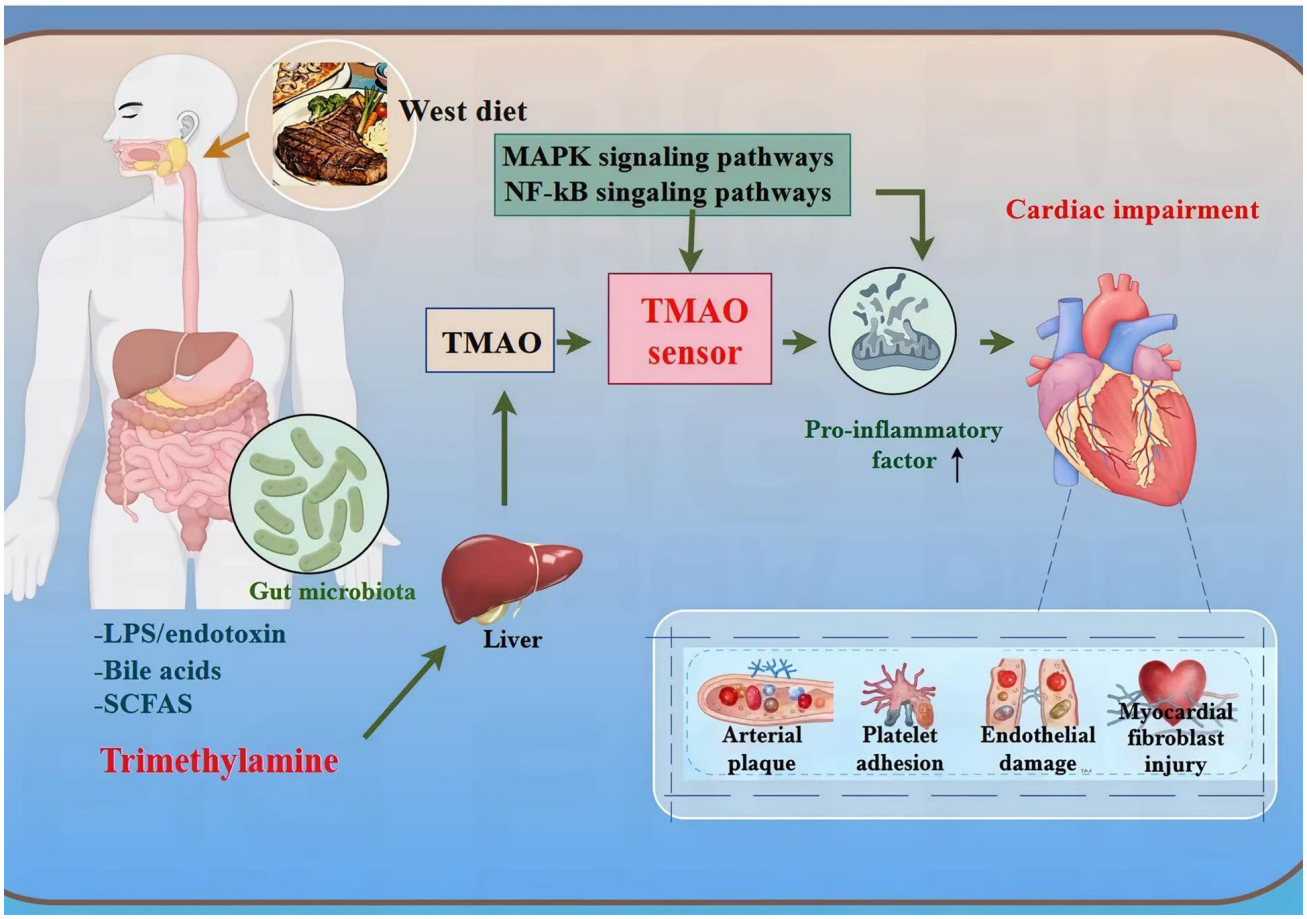
The multistep mechanism of Western diet-induced CVD. This diagram illustrates how a Western diet triggers cardiovascular disease through multiple pathways: a high-fat, high-sugar Western diet activates the insulin signaling pathway, which in turn induces fat accumulation, elevated cholesterol, high blood glucose, and immune system disorder. These abnormalities interact and collectively impact the heart, increasing the risk of cardiovascular disease.

The human gut microbiota constitutes a highly complex ecosystem comprising approximately 100 trillion microorganisms, which play a pivotal role in regulating various physiological processes of the host (Vaughn et al., 2017). Extensive research has demonstrated that dietary modifications can induce rapid compositional shifts in approximately 60% of the gut microbiota (Sonnenburg et al., 2010). In a randomized crossover study comparing WD with Mediterranean diet intervention, significant microbial changes were found in the WD group: (1) the abundance of butyric acid bacteria (e.g., Butyromonas butyricum butyricum and Butyrobacter Hadrus) species decreased; (2) The diversity of β -oxidative metabolic pathways decreases; (3) Elevated serum cholesterol levels (Gibson et al., 2007). Studies have shown that WD intake can significantly reduce the microbial alpha diversity in mouse models, which may lead to irreversible attenuation of key bacterial groups in the intestinal ecosystem (Gibson et al., 2007; see Figure 1).

### 2.1 Excessive intake of fat

The increase in fat content in the diet is one of the reasons for the changes in the composition of the intestinal flora (Jensen et al., 2018; Bier et al., 2018). Studies have shown that the Firmicutes and Bacteroidetes phyla account for approximately 90% of the human microbiome, with the remaining 10% composed of Proteobacteria, actinomycetes, Firmicutes, and Fusobacteriales (Barber et al., 2023). Therefore, the ratio of Firmicutes to Bacteroides is often used to describe the changes in intestinal flora (Djuric et al., 2018). In C57BL/6 mice fed a high-fat diet, the proportion of Firmicutes/Bacteroides was significantly increased (Djuric et al., 2018). In low-fat diet fed mice model found that the thick wall fungus door doria bacteria genera and rumen bacteria abundance increase (Zhu et al., 2016; Cui et al., 2017), and the shooter of bacteroidetes bacteria and Clinton bacteria were significantly reduce (Cui et al., 2017). A significant increase in the Firmicutes phylum will lead to a reduction in the Bacteroidetes phylum, which has an enzymatic mechanism that helps the host metabolize indigestible polysaccharides. Due to the regulatory effect of low-fat diet, the absence or reduction of Bacteroides may lead to the disorder and loss of a variety of specific microorganisms in the gut, which well explains why low-fat diet can lead to changes in the diversity of intestinal microorganisms (Djuric et al., 2018). In addition, another 6-month randomized controlled feeding intervention study for humans showed that the fecal microbiota of volunteers on a high-fat diet was significantly reduced, while the abundance of Bacteroides, *Clostridium, Bifidobacterium*, and *Lactobacillus* was significantly increased. Further research found that after 6 months of intervention, the alpha diversity of the gut microbiota in the low-fat group volunteers significantly increased, suggesting that a high-fat diet may have a negative impact on gut microbiota diversity. In addition, the activities of various bile acid (BA) hydrolases in the high-fat diet group were significantly reduced. Subsequent 16S rRNA gene sequencing analysis of the intestinal flora in the high-fat group revealed that the abundance of pro-inflammatory genes was relatively high, which might damage the intestinal barrier function (Djuric et al., 2018; Jensen et al., 2018; Barber et al., 2021).

### 2.2 High salt and sugar intake

A diet high in salt and sugar is a typical feature of WD. Due to the relatively low absorption efficiency of fructose in the human body, the intake of WD can disrupt the intestinal flora and lead to metabolic disorders (Pasini et al., 2003). Excessive intake of added sugar can alter the composition of intestinal microbiota, leading to an increase in the proportion of *Firmicutes* and Bacteroidetes, while reducing the number of beneficial butyric acid bacteria (Jensen et al., 2018). In the hypertension rat model, a high-salt diet significantly increased the levels of SCFAs, including acetic acid, propionic acid, and butyric acid, while also increasing the relative abundance of specific bacterial groups such as Clostridium, Escherichia, Clistenensaceae, and Corynebacterium. The changes in these microbial communities are directly related to the development of HTN (Svingen et al., 2013).

The Mediterranean diet is widely regarded as the opposite of WD in terms of nutritional composition. A large number of studies have shown that this dietary pattern rich in dietary fiber can not only promote the proliferation of beneficial bifidobacteria (including Bifidobacterium longidobacteria, Bifidobacterium breve, and Bacteroides), but also improve the diversity of intestinal microbes (Svingen et al., 2013). However, there are differences among various research results. Clinical trials have shown that compared with WD, the composition of the gut microbiota did not change significantly after 6 months of Mediterranean diet intervention (Whon et al., 2012). These findings suggest that short-term dietary adjustments may not be sufficient to trigger significant and lasting reshaping of the gut microbiota ecosystem (Svingen et al., 2013).

## 3 The relationship between intestinal flora disorder and CVD

Recent evidence from global case-control studies has demonstrated a significant association between gut microbiota dysbiosis and CVD pathogenesis through fecal metagenomic analysis. It is worth noting that by using shotgun metagenomic sequencing technology, it was found that there were differences in the microbial characteristics between atherosclerotic patients and the healthy control group: Collins genus was enriched in CVD patients, while Cocci genus was dominant in the healthy control group (Zuo et al., 2020). The level of glucosamine n-acetyl-6-phosphate/mannitol in patients with coronary heart disease is positively correlated with specific strains (including Clostridium perfringens HGF2, Streptococcus M334 and Streptococcus M143) (Zuo et al., 2019).

### 3.1 Short-chain fatty acids

Short-chain fatty acids (SCFAs) are volatile fatty acids containing less than 6 carbon atoms, mainly synthesized from butyric acid, propionic acid and acetic acid (Levels et al., 2003). A large number of studies have shown that SCFAs have cardiovascular protective effects. Dietary supplementation of 1% butyrate can exert anti-inflammatory effects and delay the progression of atherosclerosis by enhancing plaque stability (Laugerette et al., 2011). Propionic acid can prevent myocardial hypertrophy, fibrosis, vascular dysfunction and HTN through T-cell-dependent mechanisms (Laugerette et al., 2011). SCFAs are volatile fatty acids containing less than six carbon atoms, mainly synthesized from butyric acid, propionic acid, and acetic acid (Levels et al., 2003). A large number of studies have shown that SCFAs have cardiovascular protective effects. Dietary supplementation of 1% butyrate can exert anti-inflammatory effects and delay the progression of atherosclerosis by enhancing plaque stability (Laugerette et al., 2011). Propionic acid can prevent myocardial hypertrophy, fibrosis, vascular dysfunction, and HTN through T-cell-dependent mechanisms (Laugerette et al., 2011). Studies have shown that SCFAs can regulate vasomotor function and blood pressure levels. Specifically, acetic acid and propionic acid can alleviate systemic inflammatory responses and atherosclerotic lesions, and are independent indicators for predicting HTN (Khan et al., 2018). 16S ribosomal RNA sequencing studies found that germ-free mice transplanted with the feces of HTN patients had elevated blood pressure, and their SCFA levels and the degree of microbiota dysbiosis were significantly lower than those of the control group mice (Contreras et al., 2000). In addition, the protective effect of SCFAs on the cardiovascular system depends on their binding to specific G protein-coupled receptors (GPCRs) in the intestinal tract and systemic tissues, as well as their regulation of olfactory receptors. GPR41 and GPR43 are a group widely expressed in vascular endothelial cells, vascular smooth muscle cells (VSMCs), and immune cells (such as macrophages and Treg cells), and they are the core receptors for SCFAs to exert anti-inflammatory effects and regulate vascular tension. In the regulation of vascular tension, propionic acid and butyric acid activate GPR43 in vascular endothelial cells, promoting the activation of the phospholipase C (PLC) -IP3 pathway, increasing the intracellular Ca^2+^ concentration, and thereby activating endothelial nitric oxide synthase (eNOS), which promotes the production of nitric oxide (NO) - NO can diffuse into VSMCs. Guanylate cyclase (sGC) is activated to increase cGMP level, which leads to VSMCs relaxation and ultimately reduces peripheral vascular resistance (Mohammed et al., 2024). Clinical studies have shown that for every 1 μmol/L increase in propionic acid concentration in peripheral blood, the systolic blood pressure of patients with hypertension can decrease by 0.8 mmHg, and this effect completely disappears in GPR43 gene knockout mice (Bonnard et al., 2023). At the immunomodulatory level, after butyric acid binds to GPR41, it can recruit β-arrestin2, blocking the phosphorylation and degradation of IκBα, thereby inhibiting the transfer of NF-κB to the cell nucleus and reducing the release of pro-inflammatory cytokines (such as TNF-α, IL-6) (Pakhomov and Baugh, 2021). Furthermore, in Treg cells, butyric acid activates the PI3K-AKT pathway through GPR43, promoting the expression of fork head box protein P3 (Foxp3), enhancing the immunosuppressive function of Treg cells, and thereby alleviating the inflammatory response within atherosclerotic plaques (Mann et al., 2024). It is worth noting that the affinity of different SCfas for receptors varies: butyric acid has a higher affinity for GPR41 than propionic acid, while propionic acid has a better affinity for GPR43 than butyric acid (Xu et al., 2022). This difference may lead to the different functional emphasis of SCFA in cardiovascular protection.

### 3.2 Trimethylamine-N-oxide (TMAO)

**Figure 2:**
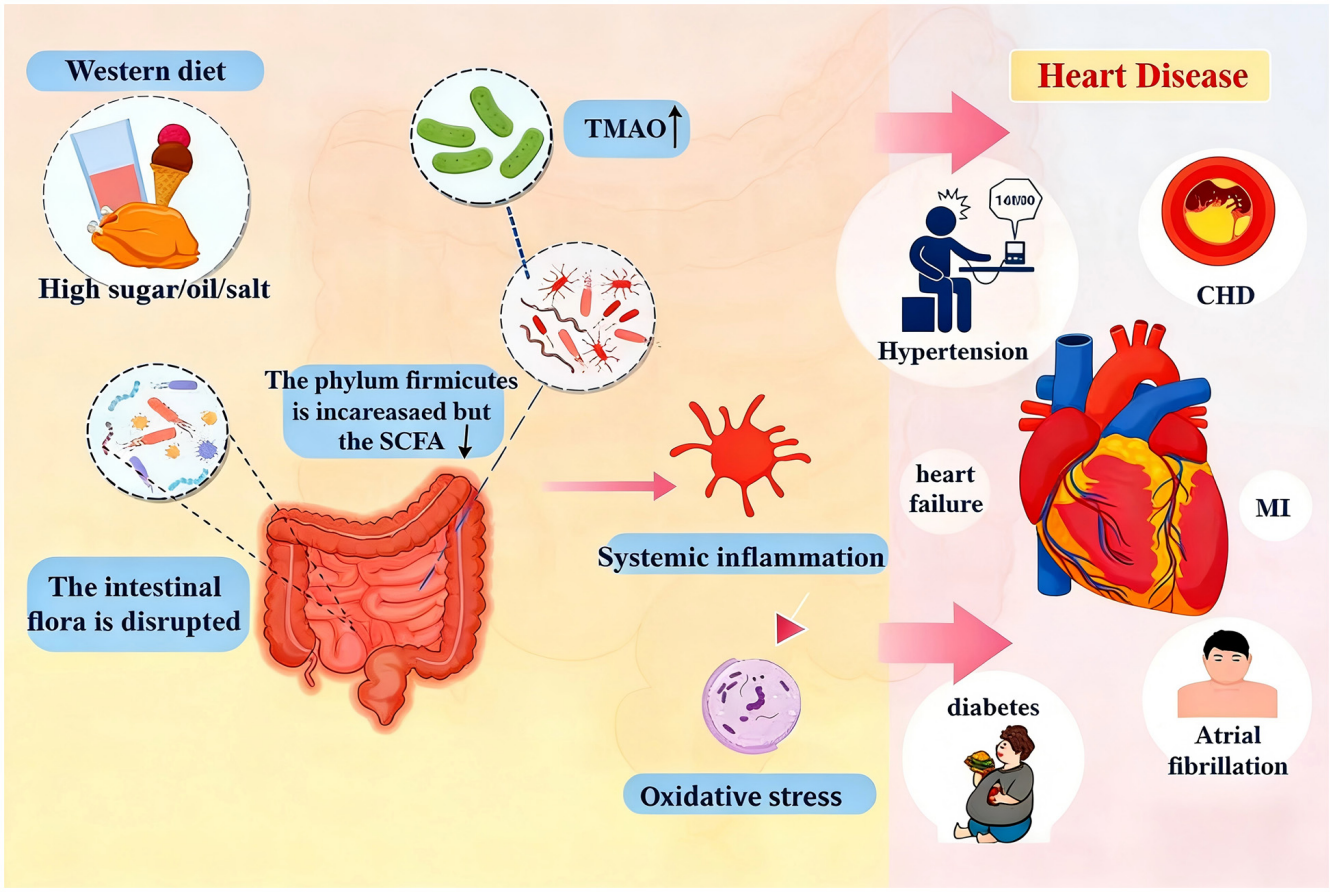
Molecular mechanism of Western diet-induced cardiac injury via the gut microbiota-liver axis. This diagram illustrates the pathogenic pathway of a Western diet: the diet acts on gut microbiota through the digestive system, producing substances like trimethylamine. Trimethylamine is then metabolized into TMAO in the liver; binding to the TMAO sensor activates the MAPK/NF-κB pathways, triggering pro-inflammatory factor release. This ultimately induces pathological changes (e.g., arterial plaques, platelet adhesion) and leads to cardiac injury.

TMAO is a small molecule metabolite synthesized by the liver from dietary precursors such as phosphatidiyl choline, choline and carnitine (Emoto et al., 2016). Increased TMAO levels due to excessive intake of choline and carnitine have been shown to have adverse effects on the host cardiovascular system (Tang et al., 2013) (see Figure 2). For instance, mice fed 1.2% choline feed had a significantly higher TMAO concentration than the control group, accompanied by a decrease in left ventricular ejection fraction (LVEF) and obvious myocardial fibrosis (Tang et al., 2013). In addition, a high-choline diet promotes the accumulation of TMAO, which prompts macrophages to transform into cholesterol-rich foam cells, thereby accelerating the process of atherosclerosis (Senthong et al., 2016). In addition to participating in lipid metabolism, TMAO can also directly enhance the overreactivity of platelets. Studies in animal models of thrombosis have shown that TMAO promotes thrombosis by accelerating platelet activation (Tang et al., 2013).

## 4 The WD-gut microbiota-metabolite axis in specific CVD subtypes

### 4.1 Coronary heart disease (CHD)

#### 4.1.1 The influence of diet on coronary heart disease

Coronary heart disease (CHD) is the leading cause of death worldwide. The 2010 Global Burden of Disease Study predicted that its incidence will increase by 50% by 2030 compared with 2010 (Lozano et al., 2012). The lifetime risk of coronary heart disease for people over 40 is approximately 49% for men and 32% for women (Mathers and Loncar, 2006). Dyslipidemia, blood glucose, blood pressure, and obesity are recognized risk factors for coronary heart disease, and dietary patterns play a key role in regulating disease risk and progression (Lloyd-Jones et al., 2002). As a well-validated statistical method, descending regression (RRR) has been widely used to explore the association between dietary patterns and coronary heart disease-related outcomes. Carotid intima-media thickness (CCA-IMT) serves as a reliable early indicator of atherosclerosis progression (Kromhout et al., 2002). A multicenter observational study found that a baseline RRR dietary pattern characterized by high intake of refined grains, processed red meat, and sugar-sweetened beverages (typical features of WD) was significantly and positively associated with CCA-IMT (Ding et al., 2025). Studies in Western regions have further confirmed that the group mainly composed of WD has a higher CCA-IMT value and the incidence of carotid artery stenosis (Nettleton et al., 2007). With the process of globalization, the WD model has also affected the Asian population: multiple studies in the Middle East have found that an increase in the intake of fat, red meat and carbohydrates, while a decrease in the intake of fruits, vegetables and green leafy vegetables, is positively correlated with the risk of coronary artery disease (Oikonomou et al., 2018). Excessive sugar intake in the diet, as a typical feature of the WD pattern, is an independent risk factor for the development of coronary heart disease (Schwab et al., 2014). A randomized controlled trial demonstrated that high sugar intake directly increases cardiovascular risk by raising blood pressure and low-density lipoprotein cholesterol (LDL-C) levels —these key factors exacerbate the risk of coronary heart disease (Fung et al., 2009). Epidemiological studies have also found that high intake of red meat and processed meat is associated with elevated serum cholesterol, which is a recognized predictor of coronary heart disease (Te Morenga et al., 2014). As early as 1908, Ignatowski observed that rabbits fed a diet high in cholesterol and saturated fat would develop atherosclerotic lesions, which proved that saturated fatty acids and cholesterol could increase serum cholesterol concentration (Steffen et al., 2005). In addition, low-density lipoprotein receptor-deficient male mice fed a low-fat diet showed significant lipid accumulation and increased mortality (McGill, 1979). Processed meat products in low-fat diets are rich in sodium, nitrates and preservatives, which can produce potential carcinogenic compounds (such as heterocyclic amines and polycyclic aromatic hydrocarbons) during cooking. These substances are epidemiologically associated with an increased risk of coronary heart disease (Bogen and Keating, 2001; Lakshmi et al., 2005; Meydani et al., 2014).

#### 4.1.2 Intestinal flora changes in patients with coronary heart disease

Breakthroughs in high-throughput sequencing technology have significantly deepened our understanding of gut microbiota dysregulation in the pathogenesis of congenital heart disease (CHD). A rigorously controlled clinical study involving 29 CHD patients and 35 healthy controls found significant microbial community alterations in CHD patients with decreased abundance of Bacteroidetes and Proteobacteria, and increased levels of *Firmicutes* and Fusobacteria (Zhu et al., 2016). Subsequent metagenomic analysis results were highly consistent with this finding (Dinakaran et al., 2014). It is worth noting that the proportion of *Firmicutes* and Bacteroides in CHD patients is increased, which is significantly positively correlated with metabolic disorders such as obesity and dyslipidemia. Mechanism studies have shown that this imbalance of microbiota promotes ectopic fat deposition by enhancing energy acquisition efficiency and improving intestinal nutrient absorption (Gostimirovic et al., 2023). FMT experiments in animal models have provided experimental evidence for the direct causal relationship between the composition of intestinal microbiota and the progression of CHD. Studies have confirmed that susceptibility to atherosclerosis can be transmitted horizontally through the gut microbiota (Zhu et al., 2016). Furthermore, clinical studies have found that compared with the healthy control group, the relative abundance of Bacteroides and *Bifidobacterium* in patients with coronary heart disease is significantly reduced, and this reduction is negatively correlated with the level of fecal lipopolysaccharide (LPS) (Manco, Putinani, and Botazzo), which is consistent with the phenomenon of elevated fecal LPS concentration in patients with coronary heart disease reported in early epidemiological data in Japan (Dinakaran et al., 2014; Cui et al., 2017).

#### 4.1.3 Metabolic mechanisms mediating CHD

The metabolic products of intestinal microorganisms, as bioactive signaling molecules, can regulate the progression of coronary heart disease. LPS, also known as endotoxin, is mainly located on the outer membrane of Gram-negative bacteria. As a potent systemic inflammation inducer, it has been recognized as a biomarker for coronary heart disease (Yoshida et al., 2018). Changes in the intestinal flora will increase the permeability of the intestinal barrier, allowing LPS to enter the circulatory system. This influx of LPS reduces high-density lipoprotein (HDL) levels, impedes cholesterol transport and promotes cholesterol deposition in the vessel wall—further increasing the risk of coronary heart disease (Saad et al., 2016). TMAO as another key microbial metabolite, has always been associated with the incidence of coronary heart disease. Multiple studies have confirmed that the concentration of circulating TMAO is positively correlated with the risk of coronary heart disease (Ding et al., 2025). TMAO is synthesized in the liver by intestinal microbiota metabolizing dietary precursors (such as phosphatidylcholine, choline, carnitine) (Cui et al., 2017). Excessive intake of choline and carnitine (commonly found in WD) leads to elevated TMAO levels, which promotes the transformation of macrophages into cholesterol-enriched foam cells, thereby accelerating the progression of atherosclerosis (Vakadaris et al., 2025). SCFAs, including acetic acid, propionic acid and butyric acid, are key mediators in the pathogenesis of coronary heart disease. The abundance of butyric acid-producing bacteria (e.g., Roseobacter intestinalis and Faecalis prausnii) is significantly reduced in patients with CHD, suggesting that gut microbiota may influence the development of CHD by modulating SCFA lineage (Tang et al., 2013). SCFAs have cardioprotective effects: for instance, dietary supplementation with 1% butyrate can reduce inflammation and delay the progression of atherosclerosis by enhancing plaque stability (Blacher et al., 2017); Propionate prevents cardiac hypertrophy, fibrosis and hypertension through T-cell-dependent mechanisms (Aguilar et al., 2014). Acetate and propionate can adjust vasomotor function and blood pressure levels, reduce systemic inflammatory response and atherosclerotic lesions, and at the same time as an independent predictor of high blood pressure—this is a significant risk factor for coronary heart disease (Bartolomaeus et al., 2019).

### 4.2 Heart failure (HF)

#### 4.2.1 Effect of diet on HF

HF is the end-stage manifestation of a variety of CVD and remains a major clinical challenge in cardiovascular care. In the late stage of the disease, HF causes low pumping efficiency or reduced output of the heart due to left ventricular dysfunction, resulting in insufficient cardiac output (unable to meet metabolic demand) and multi-organ congestion with volume overload (Oikonomou et al., 2018). A very low-calorie diet significantly exacerbates heart damage caused by transverse aortic coarctation (Steinbusch et al., 2011). A high-fat and high-sugar diet not only leads to weight gain in WD-fed mice, but also triggers unique cardiac features, including cardiac insufficiency and metabolic dysfunction (Chen et al., 2017). After 16 weeks of WD exposure, mice developed diastolic dysfunction (Gallet et al., 2016), and similar cardiac diastolic function and individual cardiomyocyte function impairment were also observed in Osaba pigs fed with WD (Nguyen et al., 2017). Approximately half of HF cases belong to HF with preserved ejection fraction (HFpEF), which is characterized by impaired diastolic function (Olver et al., 2019). The triglyceride content in the hearts of mice fed WD increased (Steinbusch et al., 2011). Long-term intake of WD can impair glucose tolerance and disrupt cardiolipin metabolism, ultimately leading to systemic metabolic dysfunction of cardiomyocytes (Vileigas et al., 2021; Ballal et al., 2010). Wd-induced obesity, dyslipidemia, and systemic insulin resistance increase the risk of HF (Ballal et al., 2010). Mechanistically, excessive intake of sugar and fat forces the heart to increase glucose uptake and overactivate insulin signaling pathways, leading to the accumulation of toxic lipid intermediates (e.g., triglycerides, ceramide) in cardiomyocytes (Procópio Pinheiro et al., 2020). Studies on transgenic mice have also shown that an increase in fatty acid uptake and storage in cardiac tissue leads to triglyceride and neuramide deposition, which is closely related to histopathological changes and impaired diastolic function (Shiojima et al., 2002; Finck et al., 2002). Fatty acids regulate cardiometabolic function and mitochondrial function by activating peroxisome proliferator-activated receptors (PPARs). However, a long-term high-fat diet (the core component of WD) can lead to excessive fatty acid oxidation (FAO), which in turn causes elevated serum TG, cardiac insulin resistance, left ventricular hypertrophy (LVH), and diastolic dysfunction (Finck et al., 2002; Yagyu et al., 2003; Burkart et al., 2007). There is contradictory evidence regarding the role of omega-3 polyunsaturated fatty acids (PUFAs) in HF: Male Wistar rats supplemented with omega-3 PUFAs can reduce stress overload induced LVH, improve diastolic dysfunction and alleviate cardiac insufficiency in the short term, but HF progression still occurs eventually (Brigadeau et al., 2007; Duda et al., 2009). This highlights the complexity of the effects of WD on HF -given its typically high omega-6 and low omega-3 PUFA content -while its role in HF risk remains controversial.

#### 4.2.2 Changes of gut microbiota in HF

The abundance of opportunistic pathogens (including *Campylobacter, Shigella, Salmonella, Yersinia*, and *Candida*) was significantly increased in HF patients (Trøseid et al., 2015). Subsequent clinical cohort studies further confirmed that the alpha diversity of the gut microbiota in HF patients was negatively correlated with the severity of cardiac dysfunction, accompanied by elevated levels of systemic inflammatory response and oxidative stress (Pasini et al., 2003). 16S rRNA sequencing was used to analyze the gut microbiota of 20 patients with reduced ejection fraction type HF (caused by ischemic or dilated cardiomyopathy), and it was found that the internal classification diversity of HF patients was significantly reduced—manifested as a decrease in the relative abundance of *Coryneaceae*, Erythemataceae, and *Ruminococcu*s (Wang et al., 2022). This discovery further confirms the association between intestinal flora imbalance and the pathogenesis of HF. The decline in microbial diversity and the enrichment of pathogenic bacteria may accelerate the progression of the disease.

#### 4.2.3 Metabolic mechanism of mediating HF

Patients with HF usually present with reduced cardiac output and peripheral circulation congestion, which in turn leads to intestinal ischemia and edema, resulting in impaired intestinal barrier function (Zhang, 2025). This barrier dysfunction promotes the entry of harmful metabolites (such as LPS) into the systemic circulation, generating pro-inflammatory stimulation, damaging myocardial function and accelerating the deterioration of HF. Metabolomics studies have revealed that the pathogenesis of HF is closely related to bile acid metabolism disorders (Maggioni et al., 2010). Therapeutic regimens that modulate bile acid metabolism, particularly ursodeoxycholic acid supplementation, have been shown to improve peripheral circulation and prevent reperfusion injury in patients with HF. Experimental studies have further confirmed that bile acids exhibit cardioprotective effects in animal models by enhancing myocardial contractility and improving hemodynamic parameters (Qu et al., 2023). As a key mediator in the pathophysiology of HF, the mechanism of TMAO has also attracted much attention. Clinical studies have shown that elevated TMAO levels are associated with left ventricular diastolic dysfunction and poor prognosis, and persistently high TMAO concentrations are positively correlated with HF mortality (Suzuki et al., 2019; Purcell et al., 2001). The triggering effect of TMAO on HF may involve multiple mechanisms, including promoting myocardial fibrosis, damaging endothelial function, and intensifying systemic inflammation—these factors collectively lead to cardiac remodeling and a decline in cardiac function. SCFAs also play an important role in the progression of HF. The number of bacteria that produce SCFAs in patients with HF (such as Faecobacter propani and *Rosaceae* genus) decreases, leading to a reduction in SCFA levels (Pasini et al., 2003). SCFAs typically have the functions of regulating the integrity of the intestinal barrier, reducing inflammatory responses and regulating blood pressure—the absence of these protective functions may further aggravate HF. For instance, butyrate enhances the intestinal epithelial barrier function by promoting the expression of tight junction proteins, while acetate and propionate alleviate systemic inflammation by inhibiting the production of pro-inflammatory cytokines such as TNF-α and IL-6. Therefore, the decreased level of the calcitonin gene (SCFA) in patients with HF may form a vicious cycle of intestinal barrier dysfunction, inflammatory response and cardiac damage.

### 4.3 Myocardial infarction (MI)

#### 4.3.1 Effect of diet on MI

Myocardial infarction (MI) is a disease caused by the accumulation of plaques in the inner membrane of arteries, which leads to blocked blood supply to the heart and hypoxia, resulting in myocardial damage. MI, as a leading cause of death worldwide, has over 140,000 cases in the United States alone each year (Cilla et al., 2016; Smit et al., 2020). Dietary habits are closely related to the risk of myocardial infarction: especially the intake of red meat increases iron load, leading to adverse cardiometabolic consequences (Poursafar et al., 2019). Epidemiological studies have shown that high red meat intake in Costa Rica is an important risk factor for myocardial infarction (Lloyd-Jones et al., 2002), and a case-control study in Iran also found a significant positive correlation between red meat intake and the risk of myocardial infarction (Lu et al., 2015). High-fat diets (HFDs, a major component of WD) exacerbate cardiovascular complications in animal models: in aged rats, a high-fat diet significantly worsens hypertensive heart disease, leading to worsening atrial and ventricular remodeling and impaired left ventricular systolic function (Albrektsen et al., 2023). High-fat diets also trigger multiple post-MI complications, including myocardial fibrosis, endothelial dysfunction, and impaired ventricular function (Salari et al., 2023). In the mouse model, the levels of arachidonic acid (AA) and thromboxane B2 (TXB2) in mice fed a HFD diet were significantly higher than those in the control group (Watanabe et al., 2012). Arachidonic acid (AA), as one of the most abundant polyunsaturated fatty acids, can trigger inflammatory reactions, and its downstream metabolite TXB2 is significantly increased in the acute phase of myocardial infarction. These findings suggest that a high-fat diet may exacerbate the inflammatory response after myocardial infarction (Zhao et al., 2020). In addition, a low-fat diet can further aggravate cardiac remodeling after myocardial infarction by inducing cardiac hypertrophy, myocardial cell apoptosis, and interstitial fibrosis, ultimately leading to heart failure (Velagaleti et al., 2008; Santos et al., 2017). Conversely, a beneficial dietary pattern can provide cardiovascular protection. A diet rich in fish, fruits, vegetables, and polyunsaturated fats was associated with a 23% reduced risk of myocardial infarction.

#### 4.3.2 Intestinal flora

Recent studies have revealed a significant association between gut microbiota composition and myocardial infarction (MI). In patients with acute myocardial infarction (AMI), the abundance of *Firmicutes* decreased, the abundance of Bacteroidetes slightly increased, and the bacterial genera such as *Macrococcus*, Butyromonas, acidophilus, and desulfurvibrio significantly increased (Shariff et al., 2017). Animal experiments confirmed reduced microbial diversity and changes in the relative abundance of key taxa in the MI model (Cui et al., 2017). Bacterial invasion and translocation play a key role in the development of MI. The levels of LPS and bacterial ribosomal DNA (rDNA) in the circulatory system of MI patients are elevated, which may affect the cardiac inflammatory response and prognosis after MI (Manco et al., 2010; Tang et al., 2019; Zhou et al., 2018). Serum LPS levels were significantly higher in patients with MI than in controls, and LPS concentrations were positively correlated with blood bacterial load—suggesting that increased intestinal permeability after MI may lead to intestinal bacteria entering the circulation (Carnevale et al., 2020). In addition, serum LPS levels are strongly correlated with the neutrophil/lymphocyte ratio (NLR), white blood cell count, and neutrophil count—these systemic inflammatory markers (Carnevale et al., 2020). The germ-free mouse experiment further verified the role of the gut microbiota in MI. The deficiency of intestinal flora significantly reduced the levels of serum LPS and inflammatory cytokines. This leads to a reduction in cardiac inflammation and an improved prognosis of myocardial infarction (MI), indicating that the translocation of gut microbiota metabolites is a new mechanism for excessive inflammation and severe myocardial injury in MI patients (Carnevale et al., 2020).

#### 4.3.3 Metabolic mechanism

Lps-mediated myocardial infarction is a key microbial metabolite driving the pathogenesis of myocardial infarction. After myocardial infarction, due to the reduction in cardiac output and intestinal ischemia leading to intestinal barrier dysfunction, LPS can enter the systemic circulation. LPS activates the Toll-like receptor 4 (TLR4) signaling pathway in immune cells and cardiomyocytes, triggering the release of pro-inflammatory cytokines such as TNF-α, IL-6, and IL-1β. These cytokines can exacerbate myocardial inflammation, promote myocardial cell apoptosis and impede myocardial repair, thereby deteriorating the prognosis of myocardial infarction (Han et al., 2021; Carnevale et al., 2020). A high-fat diet is also involved in the progression of myocardial infarction. Intestinal flora imbalance caused by high-fat diet and excessive intake of choline/carnitine can lead to high levels of TMAO, thereby enhancing platelet hyperreactivity: in animal thrombosis models, TMAO promotes thrombosis by accelerating platelet activation (Wang et al., 2011). TMAO will increase the risk of coronary artery thrombosis—a major cause of MI. In addition, a high-fat diet promotes the instability of atherosclerotic plaques by increasing macrophage infiltration and reducing collagen content in plaques, making them more prone to rupture and triggering AMI (Organ et al., 2016). SCFAs have a protective effect on MI. The reduced level of SCFA in patients with MI (due to the decrease in SCFA-producing bacteria) weakens its anti-inflammatory and cardioprotective functions. For instance, butyrate can inhibit histone deacetylases (HDACs) in cardiomyocytes, thereby reducing oxidative stress and apoptosis. Acetate and propionate can also alleviate systemic inflammation by inhibiting the production of pro-inflammatory cytokines and enhancing the function of regulatory T cells (Bartolomaeus et al., 2019; Aguilar et al., 2014). Probiotic supplements (which can promote the generation of SCFA) can reduce the blood leptin level of myocardial infarction rats by 41%, shrink the MI area by 29%, and improve the mechanical function of the heart after ischemia by 23% (Jones et al., 2014). The cardiac function of mice after MI can also be improved by enhancing the diversity of intestinal microbiota and the generation of SCFA (Moraes-Silva et al., 2017).

### 4.4 Atrial fibrillation (AF)

#### 4.4.1 Effect of diet on AF

Atrial fibrillation (AF), as the most common type of arrhythmia, is the second leading cause of death in many industrialized countries (Schnabel et al., 2015; Go et al., 2003). Over the past 20 years, the number of people hospitalized in the United States due to atrial fibrillation has increased by 60%. Predictions suggest that the prevalence could soar fivefold by 2050, potentially affecting 12 million Americans (Heidenreich et al., 2022). Dietary patterns are important modulatable risk factors for atrial fibrillation (Lloyd-Jones et al., 2002). A high-fat diet (HFD, which is a core component of WD) increases the risk of atrial fibrillation. Mice fed HFD showed a higher incidence and duration of atrial fibrillation, and the mRNA levels of pro-fibrotic markers (collagen 1, collagen 3, α-SMA) in the left atrium were significantly increased (Gronroos and Alonso, 2010; Fukui et al., 2017). This is closely related to more severe atrial fibrillation fibrosis—a key driver of atrial fibrillation. Similarly, if sheep consume a high-calorie diet for eight consecutive months, it will lead to a decrease in atrial fibrillation conduction velocity and an increase in inflammation, further promoting atrial fibrillation (Zhang et al., 2020). High-fat diet-induced weight gain is a major risk factor for atrial fibrillation: Adipose tissue accumulation promotes myocardial fibrosis through dysregulation of inflammatory mediators and adipokines, leading to atrial structural remodeling and chamber dilation (Abed et al., 2013; Sumeray et al., 1988; Echahidi et al., 2007). Long-term intake of high-fat and high-calorie diets is the main cause of obesity and another independent risk factor for atrial fibrillation. Rodent model studies have shown that long-term feeding of HFD can significantly increase body weight, increase atrial mass and promote myocardial cell apoptosis (Heidenreich et al., 2022). These changes alter the host's energy metabolism, leading to the accumulation of adipose tissue in obese animals and triggering atrial structural remodeling—making them more prone to atrial fibrillation (Meng et al., 2017). Diabetes (often caused by Verdi's disease) is also an independent risk factor for atrial fibrillation: a comprehensive meta-analysis shows that the incidence of atrial fibrillation in people with diabetes is 40% higher than that in non-diabetic populations (Jia et al., 2016). Experiments using animal models of diabetes further demonstrated that a diet high in fat, sugar, and cholesterol would exacerbate atrial structural remodeling and promote the occurrence of arrhythmia (Bell and Goncalves, 2019; Pacher et al., 1999). On the contrary, a beneficial dietary pattern can reduce the risk of atrial fibrillation. The incidence of atrial fibrillation among the lowland indigenous people of Bolivia (such as the Zimane and Moselten) is extremely low, which is attributed to their diet rich in dietary fiber, polyunsaturated fatty acids, potassium, magnesium, and selenium (Watanabe et al., 2012). The Mediterranean diet—characterized by a large intake of green leafy vegetables, fruits, fish and moderate amounts of wine—has a heart-protective effect on atrial fibrillation. The Mediterranean diet not only improves obesity, hypertension and dyslipidemia in patients, but also significantly reduces systemic inflammatory markers—all of which are related to the pathogenesis of atrial fibrillation (Rowan et al., 2021). These findings suggest that the risk of AF can be effectively reduced by modifying the diet to reduce high-fat and sodium intake and increase dietary fiber-rich foods.

#### 4.4.2 Changes of intestinal flora in AF

Intestinal flora imbalance is closely related to the onset of AF. High-throughput sequencing studies (including metagenomics and metabolomics) have found that regardless of whether patients belong to the subtype of persistent atrial fibrillation (psAF) or paroxysmal atrial fibrillation (PAF), their characteristic is a decrease in microbial diversity. In addition, the numbers of key bacterial groups such as Faecobacter, Prevotella, Alistapbacterium, Actinobacter, Sartre, and Faecobacter Plusnitz decreased significantly. The genera of *Butyricococcus, Flavobacterium*, and *Bifidobacterium* also showed a similar downward trend (Svingen et al., 2013). Among them, the symbiotic butyric acid bacteria with anti-inflammatory properties—Faecobacter Plusnitz—have attracted much attention. Its deficiency is related to the pathological mechanism of inflammation, thereby promoting the occurrence of atrial remodeling and atrial fibrillation (Zuo et al., 2019). The imbalance intestinal flora is closely related to the onset of AF. High-throughput sequencing studies (including metagenomics and metabolomics) have found that regardless of whether patients belong to the subtype of psAF or PAF, their characteristic is a decrease in microbial diversity. In addition, the numbers of key bacterial groups such as Faecobacter, Prevotella, Alistapbacterium, Actinobacter, Sartre, and Faecobacter Plusnitz decreased significantly. The genera of *Butyricococcus, Flavobacterium*, and *Bifidobacterium* also showed a similar downward trend (Svingen et al., 2013). Among which, the symbiotic butyric acid bacteria with anti-inflammatory properties—Faecobacter Plusnitz—have attracted much attention. The deficiency of Faecobacter Plusnitz is related to the pathological mechanism of inflammation, thereby promoting the occurrence of atrial remodeling and atrial fibrillation (Zuo et al., 2019). Previous studies have confirmed that the numbers of Alistroplata and Oscillobacter, which are beneficial bacteria for maintaining intestinal ecological balance and reducing cardiovascular risk, also decrease in patients with atrial fibrillation, further disrupting intestinal homeostasis (Miquel et al., 2013; Nagai et al., 2010). There are also significant changes in the enterovirus group in patients with AF: a significant increase in virus diversity, accompanied by structural changes in the enterovirus population (such as increased proportion of streptococcal phage DT1 and Pseudomonas phage), and functional imbalance of enterovirus activity, especially in the membrane structure composition and metal ion binding pathway (Barber et al., 2021). These viral changes may affect the bacterial community indirectly (such as lysing beneficial bacteria) or directly act on host cells, thereby promoting the occurrence of atrial fibrillation.

#### 4.4.3 Metabolic mechanisms mediating AF

TMAO is a key microbial metabolite driving the progression of AF. Intestinal flora can promote the conversion of dietary choline/carnitine into TMAO, which in turn accelerates the polarization of M1 macrophages and causes cell pyrosis, thus aggravating the remodeling of atrial structure (Patti et al., 2017; Yang et al., 2015). Multiple studies have confirmed a significant association between TMAO levels and the occurrence of AF (Rowan et al., 2021; Yu et al., 2018) suggesting that direct injection of TMAO into the ganglionated plexus of dogs induces atrial electrical remodeling through myocardial fibrosis and cardiac dysfunction, thereby promoting the induction and maintenance of AF. SCFAs plays a protective role on AF. The number of SCFA-producing bacteria (such as Faecobacter praxis and Streptococcus butyrate) in patients with atrial fibrillation decreases, leading to a drop in SCFA levels and weakening its anti-inflammatory and anti-fibrotic effects. For instance, butyrate effectively inhibits atrial fibrosis by suppressing the key driver of atrial collagen deposition—the TGF-β/Smad signaling pathway. Acetate and propionate also reduce systemic and atrial inflammation by inhibiting the production of pro-inflammatory cytokines such as TNF-α and IL-6 and enhancing regulatory T cell function (Barber et al., 2023; Aguilar et al., 2014). The absence of these protective effects will exacerbate atrial remodeling and increase susceptibility to AF. LPS may also be involved in the pathogenesis of atrial fibrillation. The imbalance of intestinal flora and intestinal barrier dysfunction in patients with AF enable LPS to enter the systemic circulation, activate the TLR4/NF-κB signaling pathway in atrial myocytes and fibroblasts, release pro-inflammatory and pro-fibrotic mediators, and trigger the occurrence of atrial inflammation and fibrosis, the key inducements of AF (Carnevale et al., 2020; Zuo et al., 2020). In addition, inflammation induced by LPS impairs electrical conduction in the atria, which increases the risk of arrhythmia.

### 4.5 Hyperlipidemia

#### 4.5.1 Effect of diet on hyperlipidemia

Hyperlipidemia is a metabolic disorder characterized by lipid metabolism imbalance and is a major risk factor for atherosclerotic CVD (ASCVD) (Albrektsen et al., 2023). It is defined as elevated levels of serum total cholesterol (TC), triglycerides (TG), and low-density lipoprotein cholesterol (LDL-C), while decreased levels of high-density lipoprotein cholesterol (HDL-C). Hyperlipidemia causes nearly half of global deaths (Giau et al., 2015), and its prevalence and mortality rates continue to rise (Global Burden of Disease Study Collaborators, 2017). An unreasonable diet structure, especially WD, can promote the occurrence of hyperlipidemia. High fat (21%), high cholesterol (1.5%) and carbohydrate (50%) of WD (Vasquez et al., 2012; Schierwagen et al., 2016) will accelerate key genetic factors in the lipid metabolism of apolipoprotein E (APOE) lack (Beltrán-Debón et al., 2011). APOE regulates blood lipid and lipoprotein levels through a variety of mechanisms (Giau et al., 2015), and APOE knockout mice develop severe hypercholesterolemia (“Global, regional, and country incidence, prevalence, and years lived with disability for 354 diseases and injuries, 1990–2017, in 195 countries and territories: a systematic analysis of the Global Burden of Disease Study Collaborators (2017), (2018). In WD mice, plasma TC levels were increased 3 to 7 times, and long-term exposure could increase TC levels as high as 1,200–1,400 mg/dL (Beltrán-Debón et al., 2011). Humans and animals with APOE gene defects are rich in plasma cholesterol and are more likely to develop WD and rapidly develop hyperlipidemia and atherosclerosis (Jeon et al., 2015). Phytosterols have a protective effect on hyperlipidemia: daily intake of 2 grams of phytosterols can effectively lower LDL-C levels and prevent the occurrence of hyperlipidemia. However, a typical WD contains only about 300 mg/day of phytosterols, which is insufficient to exert its protective effect (Zhang et al., 1992; Piepoli et al., 2016). Therefore, long-term adherence to WD will inevitably lead to the development of hyperlipidemia. In addition, WD can trigger oxidative stress and disrupt autophagy—a key process in lipid metabolism. In the APOE gene knockout mouse model, 7 weeks of WD feeding led to a doubling of reactive oxygen species (ROS) production in the aorta, and a more significant fourfold increase after 8 weeks (Bujak et al., 2015). ROS and other free radicals can cause imbalances in biological systems, leading to oxidative damage to cells and tissues, and increasing susceptibility to hyperlipidemia and cardiovascular complications (Judkins et al., 2010). After 12 weeks of WD feeding, oxidative stress markers in the blood and aorta significantly increased (especially in vulnerable areas), and the reduction in oxygen supply further promoted the development of hyperlipidemia (Parathath et al., 2011).

#### 4.5.2 Intestinal microflora changes in hyperlipidemia

The structure and function of intestinal flora are the key factors causing hyperlipidemia. 16S rRNA gene sequencing analysis of stool samples from children and adolescents with primary hyperlipidemia revealed a significant increase in the abundance of 36 bacterial taxa (most of which belonged to the phylum Firmicutes, especially the family of Rumen Coccaceae and Christensen), while Bacteroides and Akkermenia mucinalis were significantly reduced in samples from patients with hyperlipidemia (Manco et al., 2010). Existing studies have shown that the reduction of Bacteroides and the increase of *Firmicutes* are important factors leading to various metabolic disorders, including obesity (Gargari et al., 2018). Compared with healthy people, the abundance of probiotics (such as *Bifidobacterium, Lactobacillus*, and Faecobacter plusnitz) in fecal samples of patients with metabolic syndrome complicated with hyperlipidemia was significantly reduced (Ley et al., 2006). It is well known that these beneficial bacteria play a key role in lipid metabolism: bile salt hydrolase (BSHs) produced by Bifidobacterium and Lactobacillus can uncoupling bile acids and reduce the absorption of cholesterol in the intestine, while butyrate produced by Faecobacter Plusnitz can regulate lipid metabolism and alleviate inflammation (Ley et al., 2006). The decline in the abundance of this flora in patients with hyperlipidemia will disrupt lipid homeostasis, thereby aggravating the condition. Beneficial dietary components can improve hyperlipidemia by regulating the intestinal flora. The polyphenol components in red wine can promote glucose and lipid metabolism by increasing the abundance of Enterococcus, Prevococcus, Bifidobacterium, Bacteroides, Egeria, and Brucella, while improving endothelial function and cardiac function (Agouni et al., 2009; Moreno-Indias et al., 2016). Among which, the genus *Prevotella* regulates cholesterol synthesis by fermenting dietary fiber to produce SCFAs. Bacteroides and *Bifidobacterium* reduce cholesterol absorption through β -hydroxylase (BSH) activity (Agouni et al., 2009).

#### 4.5.3 Metabolic mechanism

Short-chain fatty acids (SCFAs) are key mediators in the regulation of lipid metabolism, and low SCFA levels in the body will accelerate the development of disease in patients with hyperlipidemia. Children and adolescents with hyperlipidemia have significantly lower levels of acetic, propionic, and butyric acid (Gargari et al., 2018). Acetic acid and propionic acid have dual regulatory effects on cholesterol metabolism: as a precursor for cholesterol synthesis in the liver, a decrease in the level of acetic acid can disrupt cholesterol homeostasis. Propionic acid, inhibit 3 - hydroxy - 3 - methyl glutaric acyl coenzyme A (HMG CoA reductase activity speed limit (cholesterol synthesis of the key enzymes) to suppress the liver cholesterol synthesis (Mohania et al., 2013). In a high-fat rat model, increased SCFA-producing bacteria significantly improved the symptoms of obesity and hyperlipidemia, which confirmed the protective effect of SCFAs (Mohania et al., 2013). In addition, TMAO and LPS are also important triggers of hyperlipidemia. The levels of TMAO and LPS in patients with hyperlipidemia are significantly elevated (Gargari et al., 2018). Among them, TMAO promotes lipid accumulation in macrophages and hepatocytes, increases the intake of LDL-C, and simultaneously inhibits cholesterol excretion mediated by HDL-C, thereby exacerbating hyperlipidemia and atherosclerosis (Organ et al., 2016). LPS activates the TLR4/NF-κB signaling pathway in hepatocytes and adipocytes, promoting the release of pro-inflammatory cytokines (such as interleukin-6, TNF-α), and further disrupting the lipid metabolism balance. For example, TNF-α can reduce the expression of low-density lipoprotein receptor in hepatocytes, thereby hindering the clearance of low-density lipoprotein cholesterol and promoting lipolysis in adipocytes, resulting in increased serum triglyceride levels (Moreno-Indias et al., 2016; Gargari et al., 2018). Regulating the intestinal flora to restore metabolic balance is an effective strategy for reversing hyperlipidemia. After supplementing garnet acid in a mouse model with a high-fructose and high-fat diet, the proportion of *Muribaculaceae* microbiota increased, the proportion of *Blautia* microbiota decreased, and the level of SCFA rose simultaneously, thereby improving the symptoms of hyperlipidemia (Queipo-Ortuño et al., 2012). Peanut polypeptides (prepared by mixed fermentation of peanut powder) can significantly enhance the diversity of intestinal flora, reduce the body weight of mice, and alleviate the negative impact of high-fat diet on lipid metabolism in hyperlipidemic mice (Mohania et al., 2013). Quercetin promotes the growth of beneficial bacteria, inhibits the reproduction of harmful bacteria, reduces the ratio of *Firmicutes* to Bacteroides, regulates dyslipidemia, and has a liver-protective effect (Ding et al., 2023; Velasquez and Katz, 2010).

### 4.6 Hypertension

#### 4.6.1 The influence of diet on hypertension

Hypertension is a major burden on global public health. Abundant epidemiological evidence suggests that WD patterns (WD), characterized by high sodium intake (Lozano et al., 2012), high saturated fat and refined sugar intake, and insufficient dietary fiber intake, contribute to the pathogenesis of hypertension through a variety of mechanisms, including immune dysregulation, elevated plasma C-reactive protein levels, and secondary systemic inflammation (Xu and Knight, 2015). High sodium intake is a typical feature of the WD pattern and the main pathogenic factor of hypertension (Rust and Ekmekcioglu, 2017). Dietary sodium exerts its hypertensive effect by regulating the composition of the intestinal flora and disrupting microbiota-inflammatory homeostasis. Epidemiological data show that the dietary pattern characteristic of WD patterns over the past three decades is strongly associated with a significant increase in refined sugar intake (Rust and Ekmekcioglu, 2017). Clinical studies have shown that after consuming sweet beverages containing glucose and fructose, cardiac rate, cardiac output and blood pressure all significantly increase within 2 h (Popkin and Nielsen, 2003). In animal models, a long-term intake of high saturated fat and sugar in the WD pattern can cause persistent systolic hypertension. These dietary patterns exacerbate aortic stiffness and inhibit endothelium-dependent vasodilation, which is a key mechanism of obesity-related metabolic and vascular dysfunction (Brown et al., 2008; Hannou et al., 2018). Transcriptome analysis of mice fed HFD diet revealed that the expansion of visceral adipose tissue (VAT) was an independent risk factor for the onset of hypertension. Visceral adipose tissue, as an active endocrine organ, can secrete pro-inflammatory cytokines (for example, factors such as tumor necrosis factor -α (TNF-α) and interleukin-6 (IL-6) can cause arterial endothelial dysfunction, promote lipid deposition, and drive fibrotic changes. These mechanisms can disrupt normal vascular homeostasis and blood pressure regulation (Padilla et al., 2015; Gibson et al., 2007). Under normal circumstances, vascular endothelium regulates vascular tension by balancing the release of vasoactive mediators, including vasodilators (such as nitric oxide NO) and vasoconstrictors (such as angiotensin II) (Su et al., 2024). In mouse models, VAT hypertrophy induced by angiotensin II receptor shows significant upregulation of Spp1, angiotensin II and Postn expression. These molecular changes are associated with impaired vasodilation function, increased vascular stiffness and enhanced fibrotic remodeling, jointly promoting the development of hypertension (Gonzalez and Selwyn, 2003).

#### 4.6.2 Intestinal flora

A meta-analysis of clinical trials has shown that probiotic fermented milk significantly reduces systolic and diastolic blood pressure in patients with hypertension (Wang et al., 2023). Comprehensive metagenomic and metabolomic analysis revealed that the microbial richness and diversity in patients with hypertension were significantly reduced (Li et al., 2017). After transplanting the fecal microbiota of hypertensive patients into germ-free mice, significant deficiencies were observed at the levels of *Anaerotruncus, Coprococcus, Ruminococcus, Clostridium*, Roseburia, *Blautia*, and *Bifidobacterium*. These findings are consistent with the results of previous metagenomic analyses (Dong et al., 2013; Li et al., 2017; Yang et al., 2015). Reverse transcription quantitative polymerase chain reaction (RT-qPCR) technology has filled the gap in the research on the distribution of intestinal flora in patients with hypertension. The positive association of rectal Bacteroides with systolic and diastolic blood pressure and the negative association of Bacteroides and *Bifidobacterium* strongly confirm the direct and significant association between gut microbiota and hypertension (Gargari et al., 2018). Animal model studies have further confirmed the imbalance of intestinal flora in patients with hypertension. All animal models of hypertension showed dysbiosis of intestinal flora, with a significant reduction in the number of SCFA-producing bacteria (Yan et al., 2017). For example, in the rat fecal model induced by chronic angiotensin II (Ang II) infusion, the number of bacteria producing acetic acid and butyrate decreased significantly (Bier et al., 2018). In the rat model of hypertensive apnea syndrome, the number of *Lactobacillus* increased, while the population of *Lactobacillus* butyrate, which converts lactic acid into butyric acid, decreased exponentially (Yang et al., 2015). The changes in these SCFA-producing bacteria lead to a decrease in SCFA levels, thereby weakening its blood pressure-lowering effect.

#### 4.6.3 Metabolic mechanism

**Figure 3:**
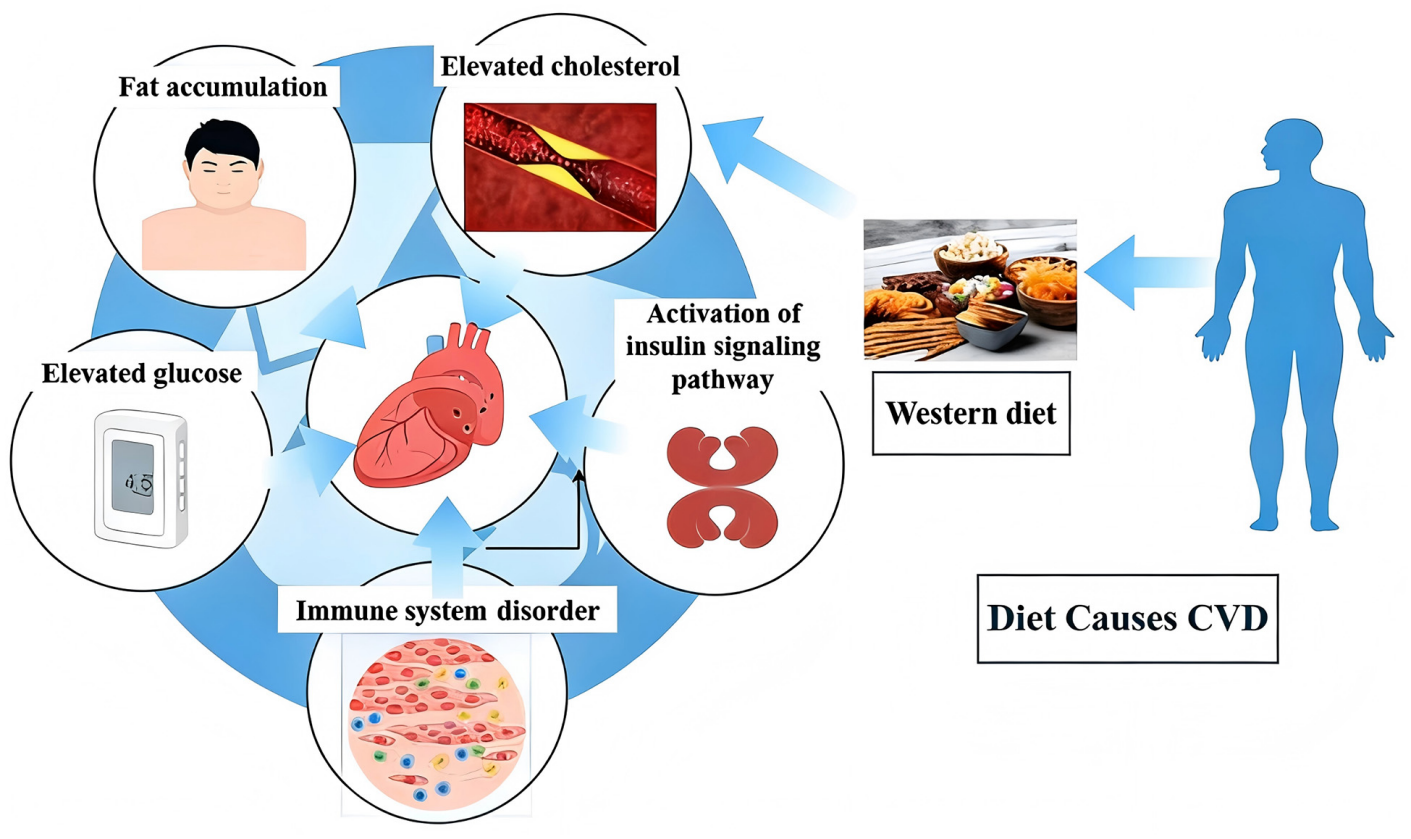
Pathological chain of Western diet (High Sugar/Fat/Salt) inducing heart disease via gut microbiota dysbiosis. This diagram illustrates the harmful pathway of a Western diet: its high sugar, fat and salt components disrupt gut microbiota (increased Firmicutes phylum, decreased SCFAs), leading to elevated TMAO, systemic inflammation, and oxidative stress. These then trigger issues like hypertension and diabetes, ultimately causing various heart diseases such as CHD, MI, and heart failure.

Short-chain fatty acids (SCFAs) are key microbial metabolites that regulate blood pressure—a decline in their levels is closely related to the onset of hypertension. Butyrate can alleviate the pro-inflammatory effect caused by LPS stimulation (Gargari et al., 2018), and effectively lower blood pressure (Wright et al., 1990). Acetate and propionate both have the effect of regulating blood pressure: acetate can enhance the production of nitric oxide (NO) in endothelial cells and promote vasodilation; Propionate inhibits sympathetic nerve activity and reduces vascular resistance (Aguilar et al., 2014). A high-fiber diet lowers blood pressure by increasing the concentrations of Bacteroides and acetate in the intestinal tract, confirming the antihypertensive effect of SCFAs (Durgan et al., 2016). LPS plays an important role in the pathogenesis of hypertension. The intestinal flora imbalance and intestinal barrier dysfunction in patients with hypertension enable LPS to enter the systemic circulation and activate the TLR4/NF-κB signaling pathway in vascular endothelial cells and smooth muscle cells. This will trigger the release of pro-inflammatory cytokines (such as interleukin-1 β, TNF-α, interleukin-6, IL-6), leading to impaired endothelial function—reducing NO production and causing vasoconstriction. LPS can also promote the proliferation and migration of vascular smooth muscle cells, leading to vascular remodeling and increased vascular stiffness, thereby further raising blood pressure (Gargari et al., 2018; Lozano et al., 2012). TMAO may also play an important role in hypertension. Elevated TMAO levels in patients with hypertension (caused by intestinal flora imbalance resulting from WD) can enhance platelet hyperreactivity and promote vascular inflammation, thereby exacerbating hypertension and its complications (Chen et al., 2025; Zhu et al., 2017). TMAO also impairs endothelial function by reducing the bioavailability of NO, further exacerbating vasoconstriction and elevated blood pressure. Increasing the level of SCFA may become a potential therapeutic strategy for blood pressure management. Studies have shown that increasing SCFA production through high-fiber diets, probiotic supplements, and prebiotic supplements has demonstrated blood pressure-lowering effects in both human and animal experiments (Bier et al., 2018). For instance, probiotic-fermented milk can lower blood pressure in patients with hypertension (Chen et al., 2025), while a high-fiber diet can reduce blood pressure by increasing SCFA levels (Durgan et al., 2016). These findings highlight the potential value of targeting the gut microbiota and SCFA metabolism in the prevention and treatment of hypertension (see Figure 3; Table 1).

**Table 1:** Changes of microbial communities in CVD.

**CVD**	**Experimental subject**	**Experimental method**	**Simplified experimental result**	**References**
CHD	Human	Rigorously controlled clinical study, microbial community analysis	*Firmicutes*/Fusobacteria increased; Bacteroidetes/Proteobacteria/Bacteroides/*Bifidobacterium* decreased; *Bifidobacterium* reduction negatively correlated with fecal LPS	Dinakaran et al., 2014; Meighani et al., 2017; Yoshida et al., 2018
HF	Human	Clinical cohort study; 16S rRNA sequencing	Opportunistic pathogens increased; gut microbiota α-diversity negatively correlated with cardiac dysfunction severity; Corynebacteriaceae/*Erythrobacteraceae*/*Ruminococcus* decreased	Pasini et al., 2003; Wang et al., 2022
MI	Human and animal models	Microbial community analysis; 16S rRNA sequencing; germ-free mouse experiment	*Firmicutes* decreased, Bacteroidetes slightly increased; *Macrococcus*/Butyromonas enriched; serum LPS/bacterial rDNA elevated; germ-free mice showed reduced inflammation and improved prognosis	Manco et al., 2010; Shariff et al., 2017
AF	Human	Metagenomic/metabolomic analysis; high-throughput sequencing	Gut microbiota diversity decreased; *Faecobacterium*/*Prevotella*/*Bifidobacterium* reduced; enterovirus diversity and phage proportion increased	Nagai et al., 2010; Barber et al., 2021
HP	Human	16S rRNA gene sequencing; fecal sample analysis	Firmicutes (36 taxa) increased; Bacteroides/*Akkermansia muciniphila*/probiotics (*Bifidobacterium*/*Lactobacillus*) decreased	Manco et al., 2010; Ley et al., 2006
HP	Human and animal models	Metagenomic/metabolomic analysis; RT-qPCR; FMT experiment	Microbiota richness/diversity reduced; *Anaerotruncus*/*Coprococcus*/Roseburia/*Bifidobacterium* deficient; SCFA-producing bacteria decreased	Li et al., 2017; Gibson and Wang, 1994

## 5 The role of gut microbiota modulation in the prevention and treatment of CVD

### 5.1 Probiotic therapy

Proper supplementation of probiotics can regulate the composition and function of intestinal flora, which has a positive impact on cardiovascular health. A meta-analysis of 846 patients with hypertension showed that the use of probiotics resulted in modest but significant reductions in blood pressure, body mass index, and blood glucose levels, highlighting their potential value in hypertension management (Choo et al., 2023). Probiotics are an inherent part of the gut microbiota, and several animal experiments have confirmed that probiotic supplementation may have therapeutic effects on CVD. For example, in a rat model of hypercholesterolemia, probiotic supplementation reduced plasma total cholesterol and triglyceride levels by 35% and 72%, respectively, while cholesterol levels decreased by 59% and significantly improved the atherosclerosis index (Gallè et al., 2020). In the apolipoprotein E gene knockout mouse model, probiotics can significantly reduce the levels of serum TMAO and cecal trimethylamine (TMA). These effects were accompanied by improvements in body weight and lipid profile optimization, as well as a delayed progression of atherosclerosis, possibly through modulation of the relative abundance of key gut microbiota families such as Tricspirillaceae, Dantocilaceae, Bacteroidaceae, and Larnaciaceae (Gibson et al., 2004; Gibson and Wang, 1994). In addition, studies have confirmed that probiotics can effectively alleviate cell apoptosis and improve depressive symptoms after MI (Pessione, 2012; Figueroa-González et al., 2011). Taken together, these findings suggest that patients with coronary artery disease (CVD) can be treated with personalized probiotic dietary supplementation to restore gut microbiota balance, thereby reducing cardiovascular risk from dysbiotic flora.

### 5.2 Dietary therapy

Multiple long-term follow-up studies have continuously confirmed that long-term dietary habits significantly affect the composition of the gut microbiota and help reduce the risk of CVD (Gibson and Wang, 1994). The Mediterranean diet (MedDiet), as a dietary pattern in sharp contrast to WD, has been proven to effectively regulate the human microbiota and has significant benefits in preventing CVD. The MedDiet diet emphasizes plant-based diets and semi-vegetarianism (Zhang et al., 2019), which can increase the dietary fiber content of the intestinal flora, raise the level of SCFA, and improve lipid imbalance through antioxidant and anti-inflammatory mechanisms. This therapy has also been proven to lower total cholesterol and low-density lipoprotein cholesterol levels, thereby significantly improving conditions such as hyperlipidemia, hypertension and coronary heart disease (Choi and Cho, 2016; Meighani et al., 2017). 16S rRNA amplification sequencing technology revealed that there was a significant difference in the relative abundance of bacterial communities between the Mediterranean diet group and the control group. It is worth noting that the number of *Butyricococcus* has increased, while the numbers of Collinia and Vironella have decreased—these microbiota characteristics are positively correlated with lower systolic blood pressure and blood glucose levels in cardiovascular patients (Gregory et al., 2015). In addition, adhering to the MedDiet can significantly increase the relative abundance of lactic acid bacteria (Gregory et al., 2015), thereby promoting metabolism, preventing gastrointestinal infections, and regulating allergic and inflammatory responses (Vrieze et al., 2012).

### 5.3 Prebiotic therapy

In 1995, Gibson's team first proposed the concept of “prebiotics,” which was further refined in 2004 to describe: “indigestible food components that selectively alter the composition or activity of the gut microbiota, thereby bringing health benefits to the host” (Gibson and Wang, 1994; Roberfroid, 2007). At present, the most common prebiotics are carbohydrate matrices. Modern research mainly focuses on fructooligosaccharides such as β -glucan, fructan and inulin (Carlson et al., 2017). With the advancement of metagenomic sequencing technology, an increasing amount of evidence indicates that prebiotics have beneficial effects on CVD (Naseri et al., 2022). This is attributed to its ability to significantly lower cholesterol levels. For instance, a study involving 30 adults with mild hypercholesterolemia revealed that after consuming 3 grams of barley β -glucan daily for five consecutive weeks, the serum total cholesterol level decreased by 2.18% (Wang et al., 2017). Prebiotics can prevent CVD through immune regulation. Their mechanisms of action include regulating the structure of the intestinal flora, influencing metabolic activities, and promoting the proliferation of beneficial bacteria. Short-chain fatty acids (SCFAs), as primary metabolites of intestinal microbiota fermentation, have been proven to improve hypertension and its related cardiovascular injuries through multiple mechanisms, such as inhibiting the renin-angiotensin system and suppressing the COX2/PGE2 pathway through the HDAC5/HDAC6-dependent pathway (Zhang et al., 2019).

### 5.4 Fecal bacterial transplantation

Fecal Microbiota Transplantation (FMT) has emerged as a new type of therapy in recent years. FMT is mainly to restore the balance of intestinal flora in patients by transplanting functional flora from the feces of healthy donors to the intestine of recipients (Zhang et al., 2018). FMT has demonstrated significant efficacy in the treatment of metabolic diseases such as inflammatory bowel disease (Gregory et al., 2015) and type 2 diabetes (Chen et al., 2023). Meanwhile, FMT also has great potential for development in the field of cardiovascular metabolic disease research. The team found a significant correlation between atherosclerotic plaque burden and plasma TMAO levels in a genetically diverse mouse population (Gregory et al., 2015). For this purpose, they conducted experiments using two different mouse strains: the C57BL/6J strain has a high TMAO production capacity and is prone to atherosclerosis, while the NZW/LacJ strain has a low TMAO production and is resistant to atherosclerosis. Subsequently, the cecal microbiota of these two strains were transplanted into apolipoprotein E-deficient mice. Subjects who received C57BL/6J cecal microbiota transplantation were found to have a significantly increased atherosclerotic plaque burden and associated TMAO levels after ingestion of a choline-rich diet. These results suggest that atherosclerosis susceptibility may be inherited through gut microbiota transplantation. Despite the demonstrated potential of probiotics and FMT in microbiota modulation of CVD, their clinical translation still faces key obstacles. Recent studies have highlighted safety concerns, such as FMT-associated infections in immunocompromised cardiac patients (Lombardi and Grossi, 2024), and inconsistent probiotic efficacy due to inadequate standardization of strain purity and viable bacteria numbers. Studies have found that only a few probiotic strains have been verified for cardiovascular benefit efficacy, and there are uncertainties in strain dosage, interaction of confounding factors and potential adverse reactions, so more comprehensive studies and randomized trials are urgently needed (Khan et al., 2024). To address these issues, emerging strategies are entering the preclinical/clinical stage: engineered probiotics (such as the E. esperance strain developed by Florida State University, which can express enzymes to oxidize trimethylamine to trimethylamine oxide), precise symbionts customized according to individual microbiota characteristics, and phage therapies targeting VD related pathogens (domestic teams have successfully treated multi-drug resistant bacterial infections using phage cocktail preparations). This provides new ideas for the intervention of CVD combined with drug-resistant bacterial infection (Zhu et al., 2023). Further research is needed in the future to improve the safety and efficacy of this therapy.

### 5.5 New directions for personalized microbiome intervention and precise regulation

In recent years, with the rapid development of precision medicine and multi-omics technologies (genomics, metabolomics, and metagenomics), personalized intestinal microbiota intervention has become an emerging focus in the field of cardiovascular disease (CVD) prevention.

From the perspective of precision nutrition and individualized probiotic strategies, there is significant heterogeneity in individuals' responses to diet and probiotics, and this difference is largely driven by the interaction relationship among genes, diet, and microbiota. Precision nutrition, by integrating multi-dimensional data such as genes, gut microbiome, and metabolic phenotypes, provides tailor-made dietary advice for individuals, and may offer better cardiovascular and metabolic benefits than traditional dietary patterns (Guasch-Ferré et al., 2025). Patients with hyperlipidemia carrying the APOE ε4 allele have a higher ratio of thick-walled bacteria to Bacteroidetes in the intestine and a lower fermentation efficiency of dietary fiber in the Mediterranean diet. However, supplementing with a complex probiotic of *Lactobacillus reuteri* and *Bifidobacterium longum* combined with a diet high in soluble fiber can reduce serum LDL-C levels by an additional 8% to 12%. In addition, metagenomic analysis of patients with coronary heart disease has shown (Muttiah and Hanafiah, 2025) that those with low abundance of Faecobacter przewali and rich TMAO-producing bacteria have a plasma TMAO level that drops by more than 30% after receiving customized live bacteria combination preparations, and the progression of atherosclerotic plaques is slowed down. In addition, the personalized diet recommendation algorithm constructed by the PREDICT study can effectively improve the metabolic indicators of people at high risk of CVD by integrating continuous glucose monitoring data and microbiome characteristics (Guasch-Ferré et al., 2025).

The next generation of intervention technologies is progressively overcoming the limitations of traditional approaches, particularly in the context of emerging therapies and ongoing clinical trials. Live biotherapeutic products (LBTs) have shown promise in enabling precise physiological regulation. For instance, research led by Academician Ge Junbo revealed that a deficiency of Bacteroides vulgatus in the gut microbiota of patients with coronary heart disease may disrupt deoxycholic acid metabolism. An engineered strain developed based on these findings has entered early-phase clinical trials, with preliminary results indicating a reduction in the risk of thrombotic events (Joo et al., 2024). In a US FDA-approved Phase I/II trial (NCT05268315), an engineered *Escherichia coli* Nissle 1917 strain effectively degraded dietary choline, leading to a sustained 40%−50% reduction in plasma TMAO levels among patients with hyperTMAOemia (Beniwal et al., 2023). This study further demonstrated that the TMAO-lowering effect of the engineered bacterium was more pronounced in individuals with high intestinal cutC gene expression compared to those with low expression, underscoring its potential for targeted therapy (Shi et al., 2024). Phage therapy has also achieved notable advancements in managing cardiovascular infections and modulating the microbiota. Clinical experience from the Berlin team at the German Heart Center indicates that a combination of intravenous and localized phage administration can effectively control chronic, recurrent infections associated with cardiovascular implants, offering a novel strategy for addressing complications caused by drug-resistant pathogens (Muttiah and Hanafiah, 2025). Interventions targeting microbial metabolites have likewise advanced significantly. A phase II clinical trial (NCT05402698) in patients with heart failure showed that oral supplementation with triglycerides increased intestinal butyrate concentrations by 2.3-fold and significantly improved left ventricular ejection fraction (Muttiah and Hanafiah, 2025). Furthermore, multicenter clinical trials on fecal microbiota transplantation (FMT) have demonstrated that oral administration of FMT capsules reduces systolic blood pressure by 4.34 mmHg in hypertensive patients on day 7 of intervention, with more sustained effects observed in elderly subgroups (Fan et al., 2025).

The interaction mechanisms among genes, diet, and gut microbiota underlie the context-specific effects observed in the prevention and treatment of cardiovascular disease (CVD). Pan et al. demonstrated that individuals carrying the Asp299Gly polymorphism in the TLR4 gene exhibit reduced intestinal barrier sensitivity to a high-fat diet, which is associated with a 28% lower risk of CVD (Poon et al., 2025). Zhernakova et al. reported that the secretory status of the FUT2 gene modulates the abundance of Bacteroidetes in the gut, thereby contributing to interindividual differences in lipid-lowering responses to high-fiber dietary interventions (Li et al., 2022). Furthermore, metagenomic analyses have shown that individuals with high intestinal abundance of the cutC gene produce 3.5 times more trimethylamine N-oxide (TMAO) upon consumption of a high-choline diet compared to those with low cutC abundance, significantly increasing the risk of coronary heart disease (Poon et al., 2025). These findings collectively support the development of personalized intervention strategies tailored to individual genetic profiles.

The integrated application of multi-omics technologies offers a novel perspective for dissecting the host–microbe–metabolite network. A 2022 study integrating transcriptomics and metagenomics revealed that gut microbiota can influence the progression of atherosclerosis by regulating hepatic expression of lipid metabolism–related genes (Qiao et al., 2023). Wikoff et al. (2009) employed a combined metabolomics and proteomics approach to identify 12 microbially derived metabolites capable of promoting acute coronary syndrome through activation of the complement-mediated inflammatory pathway. Despite these advances, personalized microbiota-based interventions still face challenges, including the lack of standardized frameworks for multi-omics data integration and insufficient evidence regarding long-term safety. Nevertheless, with ongoing technological advancements and the expansion of clinical research, such strategies hold promise as complementary approaches in CVD prevention and management, paving the way toward personalized “one-size-does-not-fit-all” cardiovascular health care (see Table 2).

**Table 2:** Key intervention clinical trials, microbiota modulation endpoints, and cardiovascular outcomes.

**Research team**	**Simplified experimental method**	**Microbiota modulation endpoints**	**Cardiovascular outcomes**	**References**
Relevant research team	Randomized controlled crossover trial of Mediterranean diet in 34 CVD patients	Increased *Butyricicoccus*; decreased *Collinsella, Veillonella*	Reduced systolic blood pressure and blood glucose in CVD patients	Gibson et al., 2007
Relevant research team	Cecal microbiota transplantation from C57BL/6J/NZW/LacJ mice to APOE-deficient mice	Transmit donor microbiota characteristics	Increased atherosclerotic plaque burden and TMAO in C57BL/6J donor group; confirmed microbiota-inherited atherosclerosis susceptibility	Gregory et al., 2015
Relevant research team	*Lactobacillus reuteri* + *Bifidobacterium* longum + high soluble fiber diet in hyperlipidemic patients with APOE ε4 allele	Reduced *Firmicutes*/Bacteroidetes ratio; improved fiber fermentation efficiency	Additional 8%−12% reduction in serum LDL-C	Guasch-Ferré et al., 2025
Relevant research team	Early clinical trial of engineered strain for CHD patients with insufficient Bacteroides vulgatus	Optimize Bacteroides vulgatus abundance; improve deoxycholic acid metabolism	Preliminary reduction in thrombotic event risk	Muttiah and Hanafiah, 2025
Relevant research team	Engineered *Escherichia coli* Nissle 1917 intervention in hyper-TMAOemia patients	Degrade dietary choline; regulate TMAO-related microbiota function	Sustained 40%−50% reduction in plasma TMAO (more effective in high cutC expression individuals)	Beniwal et al., 2023
Relevant research team	Intravenous + local phage therapy for chronic recurrent cardiovascular implant infections	Target drug-resistant bacteria; regulate microbiota balance	Effectively controlled infections; new strategy for drug-resistant bacteria-related complications	Muttiah and Hanafiah, 2025
Relevant research team	Oral triglycerides supplementation in heart failure patients	Increase intestinal butyrate concentration; balance microbiota metabolites	2.3-fold increase in intestinal butyrate; improved left ventricular ejection fraction	Muttiah and Hanafiah, 2025
Relevant research team	Oral FMT capsule intervention in hypertensive patients	Restore microbiota balance; increase beneficial bacteria abundance	4.34 mmHg reduction in systolic blood pressure on day 7 (sustained benefits in elderly)	Muttiah and Hanafiah, (2025)

## 6 Conclusion

This study systematically examines the intricate associations among the WD, gut microbiota, and CVD, offering a comprehensive and in-depth perspective to enhance understanding of CVD pathogenesis and inform the development of preventive and therapeutic strategies. The WD is characterized by high intake of sugar, fat, and salt, along with low fiber consumption. It not only directly disrupts metabolic homeostasis but also indirectly promotes the onset and progression of CVD through dysregulation of gut microbial balance. These adverse effects are observed across various CVD subtypes, including coronary heart disease, heart failure, hyperlipidemia, myocardial infarction, atrial fibrillation, and hypertension. Notably, gut microbiota and their metabolites play a pivotal regulatory role in these processes. A complex bidirectional relationship exists between gut dysbiosis and CVD: alterations in gut microbiota can influence cardiovascular structure and function via multiple pathways, while the development of CVD further modifies microbial composition and activity.

Specifically, WD-induced gut microbiota disruption exhibits a “layered effect”: excessive fat intake—particularly saturated fats—increases the Firmicutes-to-Bacteroidetes ratio and reduces butyrate-producing bacteria such as ^*^Butyrivibrio^*^; high dietary salt elevates the abundance of genera such as ^*^Enterococcus^*^ and ^*^Escherichia^*^, which are directly implicated in hypertension pathogenesis. High sugar consumption impairs intestinal fructose absorption, compromises α-diversity of the microbiota, and concurrently suppresses the β-oxidation metabolic pathway and bile acid hydrolase activity, thereby exacerbating microbial metabolic imbalance. This dysbiosis subsequently mediates CVD development through microbial metabolites: WD enhances the production of trimethylamine N-oxide (TMAO), promoting foam cell formation and platelet activation, thus accelerating atherosclerosis. It also diminishes short-chain fatty acid (SCFA) production, weakening their anti-inflammatory and antihypertensive effects mediated via GPR41/GPR43 receptors. Furthermore, disruption of the intestinal barrier permits translocation of lipopolysaccharide (LPS) into systemic circulation, activating the TLR4/NF-κB signaling pathway and triggering systemic inflammation and vascular endothelial injury. Importantly, these metabolites exhibit differential impacts across CVD subtypes. For example, in heart failure, elevated TMAO levels correlate with left ventricular diastolic dysfunction, whereas in atrial fibrillation, LPS contributes to atrial fibrosis.

From a clinical standpoint, modulation of gut microbiota presents a promising avenue for CVD prevention and treatment. Interventions such as probiotics, prebiotics, dietary modifications, and fecal microbiota transplantation (FMT) have demonstrated potential in restoring gut microbial equilibrium and improving the intestinal microenvironment, thereby offering novel strategies to mitigate CVD risk. However, these approaches remain largely in the investigative phase, necessitating further large-scale randomized controlled trials and long-term follow-up studies to establish their safety, efficacy, and reproducibility.

Despite significant advances, several critical questions remain unresolved. The causal nature of the relationship between gut microbiota and CVD has not been fully established, with many current conclusions derived from observational data lacking validation through interventional trials. Moreover, individual variability in response to microbiota-targeted interventions poses challenges for broad application. The feasibility and methodology of personalized microbiota-based therapies require further investigation. Future research should prioritize elucidating the underlying mechanisms linking gut microbiota and CVD, refining precision intervention strategies, and generating robust evidence to support clinical translation.

In conclusion, continued exploration of the interplay among WD, gut microbiota, and CVD holds promise for achieving breakthroughs in cardiovascular disease prevention and management, ultimately contributing to improved human cardiovascular health through innovative scientific insights and therapeutic approaches.
